# Adherence to oral anticancer treatments: network and sentiment analysis exploring perceived internal and external determinants in patients with metastatic breast cancer

**DOI:** 10.1007/s00520-024-08639-4

**Published:** 2024-06-25

**Authors:** M. Masiero, G. E. Spada, E. Fragale, M. Pezzolato, E. Munzone, V. Sanchini, R. Pietrobon, L. Teixeira, M. Valencia, A. Machiavelli, R. Woloski, C. Marzorati, G. Pravettoni

**Affiliations:** 1 Department of Oncology and Hemato-Oncology, University of Milan, Milan, Italy; 2 Applied Research Division for Cognitive and Psychological Science, IEO, European Institute of Oncology IRCCS, Milan, Italy; 3 Division of Medical Senology, IEO, European Institute of Oncology IRCCS, Milan, Italy; 4 SporeData Inc, Durham, NC USA

**Keywords:** Metastatic breast cancer, Barriers, Therapy

## Abstract

**Purpose:**

Adherence to oral anticancer treatments (OATs) is a critical issue in metastatic breast cancer (MBC) to enhance survivorship and quality of life. The study is aimed to analyze the main themes and attributes related to OATs in MBC patients. This research is part of a project titled “*Enhancing Therapy Adherence Among Metastatic Breast Cancer Patients*" designed to produce a predictive model of non-adherence, a decision support system, and guidelines to improve adherence to OATs.

**Methods:**

The study consists of an exploratory observational and qualitative analysis using a focus group method. A semi-structured interview guide was developed to handle relevant OAT themes. Wordcloud plots, network analysis, and sentiment analysis were performed.

**Results:**

Nineteen female MBC patients participated in the protocol (age mean 55.95, *SD* = 6.87). Four main themes emerged: (theme 1) individual clinical pathway; (theme 2) barriers to adherence; (theme 3) resources to adherence; (theme 4) patients’ perception of new technologies. The Wordcloud and network analysis highlighted the important role of treatment side effects and the relationship with the clinician in the modulation of adherence behavior. This result is consistent with the sentiment analysis underscoring patients experience fear of issues related to clinical values and ineffective communication and discontinuity of the doctor in charge of the patient care.

**Conclusion:**

The study highlighted the key role of the individual, relational variables, and side effects as internal and external determinants influencing adherence to MBC. Finally, the opportunity offered by *e*Health technology to connect with other patients with similar conditions and share experiences could be a relief for MBC patients.

## Introduction

A growing body of evidence has shown that adherence to oral anticancer treatments (OATs) is a critical issue during the breast cancer survivorship trajectory[1, 2]. Medication non-adherence is considered a psycho-social, physical, and economic burden in the metastatic breast cancer (MBC) population[3, 4]. When not promptly recognized and treated, the risk of non-adherence irreversibly reduces the effectiveness of the OATs, negatively impacting health status and survival rate[5, 6]. In particular, non-adherence to OATs leads to a worse quality of life (QoL), a significant decline in health status[7], and an increased mortality rate among non-adherent patients[8] and causes huge annual economic costs for the healthcare system. Accruing evidence highlighted that 50–75% of women diagnosed with breast cancer do not complete OATs over 5 years [9–12]. The mechanisms behind the adherence behaviors can be traced back to socio-demographic, educational, economic, psychological, cognitive, disease-related, behavioral, and system-related [13]. Overall, forgetting to take medicines and not being able to tolerate the side effects of drugs are among the most common causes of non-adherent behaviors [7]. From a theoretical point of view, medication adherence is a process that refers to the act of patients taking their treatments according to the prescribed instructions of the healthcare provider. This definition of medication adherence involves different phases, respectively: initiation, implementation, and discontinuation. Coherently, with this definition, a non-adherence behavior may appear in each of the previous phases [14].

Some studies in MBC patients have identified [15–17] explicit and implicit drivers of the decision to take or refuse the prescribed medications. For example, according to Yerrapragada and colleagues [17] factors predicting non-adherence included age (55 years or older) at the treatment starting date and those with a recent history of lymphatic nuclear medicine, radiation oncology, or arterial surgery [18]. Furthermore, the side effects of OATs were indicated as a great adherence barrier for breast cancer survivors [19]. Moreover, several studies have shown that it is difficult for patients to discuss their needs related to the disease and the treatments with the clinician [20–24], thus risking unintentionally being neglected by the health professionals themselves. Scientific evidence has strengthened the role of adherence in the success of treatment, and several studies have focused on possible strategies and interventions to facilitate proper pharmacological intake[9, 25].

Despite methodological discrepancies that hinder the merging of results, all studies agree that it is essential to recognize psychological, clinical, and contextual factors that exacerbate or increase adherence to therapy [19, 25–27]. Moreover, studies have highlighted the importance of identifying patients at risk of non-adherence and providing tailored intervention. Currently, *e*Health technologies have given the opportunity to create risk-predictive models [26, 28]. These models identify both internal and external adherence factors, allowing for early intervention and personalized patient support. This technological advancement enhances individual care and contributes to a deeper understanding of health behaviors, potentially leading to more effective, personalized healthcare strategies [29]. For instance, machine learning tools have been developed to support clinicians in assessing patient risk of emergency department visits [30], as well as outcomes such as mortality and complications [31]. However, few studies have evaluated the impact of computer-based tools, such as risk prediction applications on adherence to cancer therapies [29].

Only a few studies have examined the internal and external determinants of non-adherence to OAT in the MBC population [7, 32, 33]. For example, Conley and colleagues [32] studied intrapersonal and interpersonal factors associated with adherence to CDK4/6 inhibitors using a social ecologic approach. The results of this study highlighted non-adherence to OAT as a complex and multilevel phenomenon in which distinctive factors contribute to the definition of adherence behavior. MBC patients often find themselves having to manage a significant number of pills (and undergoing multiple lines of treatment), resulting in a high pill burden and polypharmacy [33]. It is noteworthy that, despite this, the treatment is primarily palliative rather than curative for most of these patients [34]. The symptoms and treatment burdens experienced by MBC patients diverge from patients with localized disease. Further, patients grappling with MBC often contend with both the direct signs of cancer and the side effects arising from therapies [35]. Thus, the decision to take a medication is a consequence from a set of determinants occurring over time, and it cannot be reduced to a single determinant. Likewise, determinants associated with non-adherence differ among sub-groups of patients [19]. Given the theoretical context outlined above, the current study explored internal and external determinants related to adherence behavior in patients with MBC and “if” and “how” *e*Health technologies might foster adherence using network and sentiment analysis. More in detail, this qualitative study aimed to explore patient perception about the MBC disease and its treatments, barriers, and resources related to the adherence to the OATs and to understand how *e*Health technologies might foster adherence to OATs and how it should be designed to empower patients with cancer during the disease trajectory. In particular, the patient’s perception of risk-predictive models and decision support systems has been analyzed. This study used an exploratory and qualitative analysis of the main themes and attributes related to adherence to OAT.

## Methods

### Study design

A qualitative observational study aimed to explore internal and external determinants related to adherence in patients with MBC and patient’s perception about the use of the *e*Health technologies in clinical practice, such as risk predictive models and shared decision support systems. Specifically, informational needs, treatment management, resources, and barriers relating to the OATs were explored. This qualitative observational study is part of an international Project titled “*Enhancing Therapy Adherence Among Metastatic Breast Cancer Patients*" (Pfizer Project—Tracking Number 65080791) designed to produce a predictive model of non-adherence and a decision support system (named TREAT acronym “TREatment Adherence SupporT”), and guidelines to improve adherence to OATs among MBC patients[26]. The Institutional Review Board of the European Institute of Oncology (IEO) approved the study in June 2022 (R1508/21-IEO 1594). All participants provided informed consent before implementing the study protocol. The study was conducted according to the Helsinki Declaration.

### Participant selection

Nineteen female patients diagnosed with metastatic breast cancer (currently under treatment) were enrolled using a convenience sampling strategy. Patients were referred by the oncologist of the Division of Senology, at the European Institute of Oncology, and subsequently contacted by the psychologist in charge of data collection, who presented the study in detail and requested consent. The following inclusion and exclusion criteria were defined. *Inclusion criteria*: being older than 18 years; having metastatic breast cancer; having an Internet connection and a PC or Tablet; and signing the informed consent. *Exclusion criteria*: the presence of psychiatric or neurological pathology; the presence of medical or oncological conditions other than metastatic breast cancer.

### Procedure

The focus groups were run according to the guidelines for qualitative research [36]. Three researchers and psychologists conducted the focus groups, one as principal moderator and two other co-moderators (moderators 2 and 3), with no hierarchical relationship to the participants. The enrolled patients are divided into 4 focus groups (about 4–5 participants per group). The duration of each focus group was approximately 60–90 min to avoid excessive fatigue and cognitive burden and to maintain attention on the issues discussed until achieving saturation. The focus groups were accompanied online using the Zoom platform to facilitate patients’ participation. All sessions were digitally recorded, and files were transcribed verbatim by three researchers and clinical psychologists. A brief semi-structured interview guide, with open questions that cover the main themes, was developed according to the primary scientific literature and after an internal evaluation conducted by an interdisciplinary committee[37]. Data collection was continued up to the point of saturation.

### Data analysis

Descriptive statistics were performed to depict the features of the sample. Emerging patterns were coded by research assistants GES, EF, and MP and cross-checked by the senior researcher, MM. Identified coding discrepancies were resolved through discussion. As new codes were created, previous interviews were re-coded to reflect these changes. All emerging themes were derived from the data. After the process of interview coding using colors to characterize thematic areas, tables were constructed for each focus group to organize the information extracted from the interviews. The tables consisted of the following items: patient ID, patient statements, statement keywords, categories, subthemes, and the themes referenced by the corresponding color code numbers. The patients’ statements were read several times to understand which sub-theme and category they applied to. With this contextualization, the keywords were identified and entered into the table for use in the next data analysis stage of data analysis. Before proceeding with the analyses, each selected keyword was reviewed by a second analyst to ensure that it accurately represented the content of the respondent’s statements.

We analyzed the data by combining all focus groups and then dividing them according to each of the four themes mentioned in the data collection section*.* The first step was to clean and lemmatize/tokenize all the textual data. We then used social network analysis to discern emerging themes within each thematic area, which was used to understand the relational patterns among keywords (the emerging themes) and the categories, as well as between the text and the emerging themes. This was accomplished by examining theme associations’ frequency, strength, and directionality; providing a relational map of each theme; and giving us the capacity to delineate thematic clusters and their category affiliations. The network analysis was also employed to elucidate the connections between patients and categories and between patients and emerging themes. These additional analyses further enhanced our understanding of the relational structure within the thematic areas by allowing us to see which emerging themes or categories had the most interplay between patients. Finally, sentiment analysis was carried out utilizing natural language processing (NLP) software such as spaCy to determine the sentiment of sentences that contained the relevant keyword. The sentiment itself was determined using the FeelIT Python package. This package was trained based on manually annotated tweets in Italian, which characterized the tweet in one of four basic emotions—anger, fear, joy, and sadness. The machine learning model was then trained using the UmBERTo language model, a subset of BERT, and trained only on Italian corpora. We generated a sentiment analysis distribution plot for each patient, category, and theme using the sentiment derived from the text data.

Further, we did a Circos plot for emotions across the defined categories, as well as for emotions and their relationship with individual patients. Circos plots provide an effective graphical representation of multivariate data, aiding in interpreting complex interconnections within the data. Plots were also performed for categories and patients divided by theme, giving us insight into the prevalent emotional association for each thematic area. Wordcloud plots were also generated for the text data for each theme and all themes combined. Wordcloud plots allow us to discern the words that appear more frequently in the text, which give us insight into the most prevalent words in the conversations and can be used to infer the convergence of topics in the text.

## Results

### Descriptive analysis

Nineteen patients diagnosed with MBC (*M*_age_ = 55. 95; *SD*_age_ = 6.87 min. 46–max 70) with different metastasis localizations were involved (Table 1). A total of 42.1% (8) of the patients had no familiarity with breast cancer, while 52.6% (10) had a familiarity with first grade and 5.3% (1) of second grade.

**Table 1:** Clinical information about metastasis localization, type of surgery, and anticancer treatments

Metastatic localization	% (*n*)	Surgery	%(*n*)	OATs	%(*n*)
Bone	21.1 (4)	Biliteral mastectomy	10.5 (2)	Abemaciclib	5.3 (1)
Bone and lymph node	5.3 (1)	Mastectomy left breast	31.6 (6)	Capecitabine	5.3 (1)
Liver	5.3 (1)	Mastectomy right breast	21.1 (4)	Capecitabine, vinorelbine	5.3 (1)
Liver and bone	10.5 (2)	Radicalization	5.3 (1)	Exemestane, everolimus	5.3 (1)
Lung	15.8 (3)	Quadrantectomy left breast	15.8 (3)	Letrozole, abemaciclib	5.3 (1)
Lung, bone, and lymph node	5.3 (1)	Quadrantectomy right breast	5.3 (1)	Letrozole, palbociclib	5.3 (1)
Lung and liver	5.3 (1)	No	10.5 (2)	Letrozole, ribociclib	10.5 (2)
Lung and pleural	5.3 (1)	–	–	Olaparib	5.3 (1)
Lymph node	5.3 (1)	–	–	Palbociclib	10.5 (2)
Pleural	15.8 (3)	–	–	Ribociclib	21.1 (4)
Skin	5.3 (1)	–	–	Talazoparib	5.3 (1)
–	–	–	–	Tamoxifen	5.3 (1)
–	–	–	–	Taxol	5.3 (1)
–	–	–	–	Vinorelbine, Endoxan, capecitabine (VEX)	5.3 (1)

#### Barriers, roadblocks, and resources to the adherence to OAT

The following four themes emerged from the focus groups: theme 1—individual clinical pathway; theme 2*—*barriers to adherence to OATs; theme 3*—*resources to fulfill adherence to OATs; theme 4—patients’ perception of new technologies supporting adherence to OATs (Tables 2 and 3)*.*

**Table 2:** Identified themes

Theme	Patients’ quotation
Individual clinical pathway	Quote 1. “I’ve been undergoing oral anticancer therapy for 8 years. (…) My case was a bit complicated, it was triple-negative breast cancer with lung and liver metastases. Then, there was a proposal for therapy options; the doctor suggested several possibilities, and I chose the one that allowed me to stay at home with my children. (…) They adjusted it to be less toxic.” Quote 2. “The current therapy (…) has fewer negative effects. The disease is under control, but the side effects are better than the previous one. And the previous one was also more laborious to manage. (…) So, the side effects are somehow manageable.” (English)
Barriers in the adherence to OATs	Quote 1. “I’d prefer to have only one oncologist following my case. (…) I prefer having some sort of security, knowing I only talk to him (…) Instead, having different people around makes me anxious because, although I know I can email or contact them if needed, I don’t feel reassured (…) So, I’d prefer just one person.” Quote 2. “Last year, during the lockdown, I had a problem; I had bleeding, and that scared me even more. Forgetting to take the pill… At that moment, the previous therapy I was on caused issues, and I wanted to stop it but didn’t know whom to turn to. (…) I got very demoralized and wanted to give up everything, who cares… I mean, I would have stopped taking the pill I was on, but then later.”
Resources to fulfill adherence to OATs	Quote 1. “I think it was crucial for me to have regular visits and check-ups. So, coming to the hospital for visits was important to me because it feels like you’re being followed. You know that whatever happens, you have a reference from the oncologist who last examined you. This regularity gave me enough serenity.” Quote 2. “I think that having an assistance from a psychologist could be very useful. Each of us has different aspects to address. Each person is different, so while I might overlook some things, another person might ruminate on them. Therefore, someone who has a conversation with you to understand your problems, issues and organize your care process could be very important.”
Patients’ perception of new technologies in supporting adherence to OATs	Quote 1. “Technologies (communities) (…) could lead to confrontation, bringing in experiences of people who have followed our therapy before us, so it can actually be a support.” Quote 2. “Having a tool available that when you need it, even being told, ‘It’s normal,’ or having support if you haven’t slept all night and have been with your thoughts (…) could be helpful. Also, I don’t think it’s easy to provide support to all patients undergoing treatment (…) from a psychological standpoint. And then, for me, concerning medications, if there are ways of intake that can help get through the days.”

**Table 3:** Identified sub-themes

Theme	Sub-Themes
Individual clinical pathway	Clinical path; side effects; lifestyle; shared decision-making; dose adjustments; treatment management; healthcare professional’s support; trust; gratitude
Barriers in the adherence to OATs	Side effects; going to the hospital; clinical values; negative lifestyle; patient-physician relationship; psycho-emotional distress; lack of perseverance; misunderstanding; other issues
Resources to fulfill adherence to OATs	Remote consultations; patient-physician relationship; wellbeing; nutrition; physical activity; positive lifestyle; treatment adjustment; support; medical checks; side effects re-evaluation; coping strategies related to medical intake; cancer-related coping strategies; positive treatment management; information
Patients’ perception of new technologies in supporting adherence to OATs	Tailored tool; support/utility; patient-physician relationship; coping strategies; sense of community/social support; sharing positive experiences; information/educational content; reminder; doubts/skepticism

### Wordcloud analysis

**Fig. 1:**
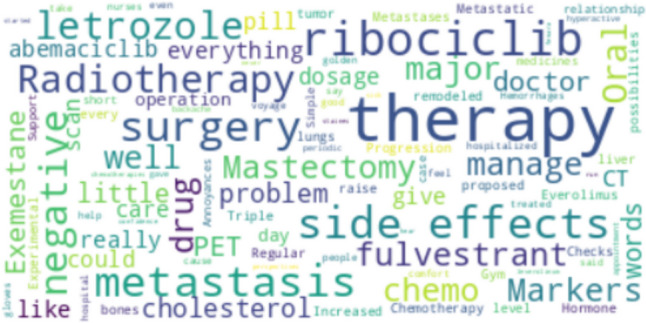
Wordcloud is based on the most recurring words on theme 1—“individual clinical pathway.” In the Wordcloud, words are displayed in different sizes based on their frequency of occurrence. More prominent words indicate a higher frequency of occurrence, while smaller words indicate a lower frequency

**Fig. 2:**
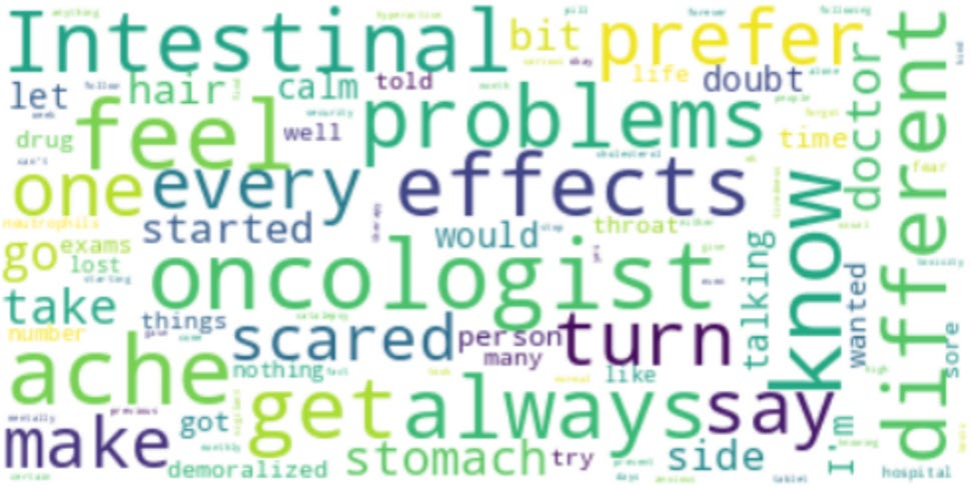
Wordcloud is based on the most recurring words in theme 2—“barriers in the adherence to OATs”

Of the nine existing sub-themes within theme 1—“individual clinical pathway,” the most frequent words belonged to two sub-themes, namely, *“*clinical course” and “side-effects,” indicating that these sub-themes are the most significant in the MBC patients’ experience of OATs. Notable terms that stand out prominently in the word cloud included different elements of the cancer pathway (therapy [*n* = 16], metastasis [*n* = 6], specific drugs (respectively, ribociclib [*n* = 6] and letrozole [*n* = 4]), and surgery [*n* = 4]) (Fig. 1). The most frequent words within theme 2*—*“barriers in the adherence to OATs” were as follows: “side effects” (*n* = 4), “stomachache” (*n* = 3), “problems” (*n* = 4), “lost hair” (*n* = 3). The higher frequency of these terms suggested the critical role of the specific type of side effects and psychological reactions as potential barriers to regular intake of the prescribed therapy. Further, the terms “physicians” (*n* = 3) and “oncologist”([*n* = 2) are present in the context of the patient-doctor relationship, especially concerning ineffective communication and continued changing physicians through clinical follow-ups (Fig. 2).

**Fig. 3:**
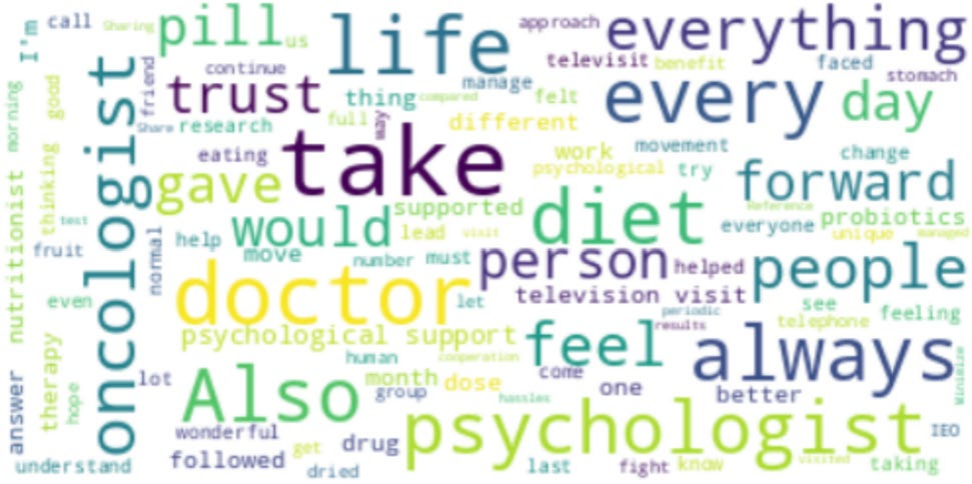
Wordcloud is based on the most recurring words on theme 3—“resources to achieve adherence to OATs.”

The key terms central to theme 3*—*“resources to fulfill adherence to OATs” highlighted possible internal and external resources patients use to increase adherent behavior (Fig. 3). In particular, the terms with a higher frequency of occurrence have been “physicians” (*n* = 6) and “psychologists" (*n* = 5) suggesting the pivotal role recognized by the patients in supporting adherence to OATs. Besides, the analysis highlights common cancer-related coping strategies, such as “moving forward despite difficulties” and “adopting healthy lifestyles,” such as “having a more active life.” Another relevant term within this theme is “diet” (*n* = 5), which refers to dietary adjustments that might help with treatment adherence to OATs, fostering overall physical well-being and counteracting the disease. Remote clinical consultations (the use of mobile applications or telemedicine) were also frequently mentioned as possible helpful aids in bolstering adherence.

**Fig. 4:**
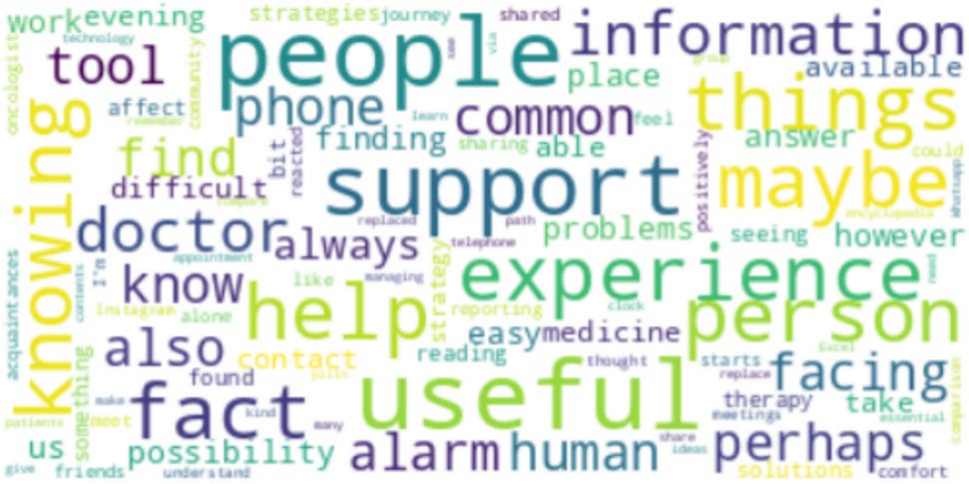
Wordcloud is based on the most recurring words on theme 4—“patients’ perception of new technologies in supporting adherence to OATs.”

The Wordcloud in Fig. 4 visually represents the most recurring words related to theme 4—“patients’ perception of new technologies in supporting adherence to OATs.” This Wordcloud analysis provides a concise yet informative overview of the human and technological resources that can support adherence. In particular, the most common words were “phone” (*n* = 3), “doctor” (*n* = 3), “person” (*n* = 3), and “support tool” (*n* = 2) and verbs related to information or knowledge such as “to know” (*n* = 3), “to find” (*n* = 3), and “to see” (*n* = 2). These nouns and verbs highlighted that new technological tools might permit oncologists and psychologists to seek supportive information quickly, improving the sense of community and social support to help each other.

### Network analysis

**Fig. 5:**
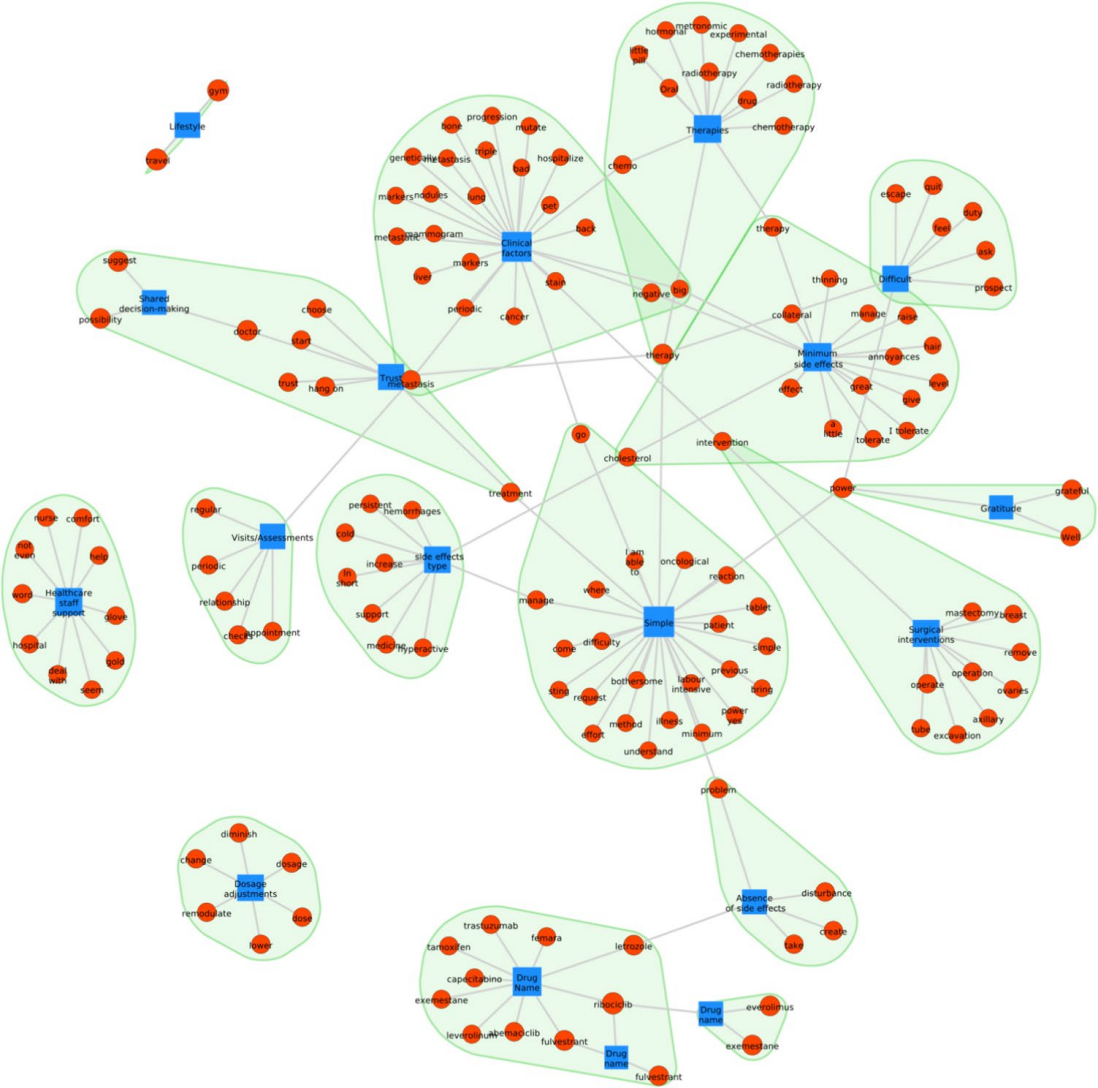
Network analysis showing the connection and recurrence between the main categories (blue) and the keywords (red) in theme 1

Clinical factors, therapies, trust, simplicity, types of side effects, and minimal side effects are the central categories of theme 1, which concerns patients’ experiences with OATs. These elements are part of the subthemes in Theme 1: patient clinical pathway, side effects, treatment management, and trust in the treatment. The centrality of these categories in the patient’s experience of OATs underscores the importance of the type and intensity of side effects, the ease of treatment management, and trust in the treatment as potential determinants of adherence to OATs. The set of keywords associated with each category forms a cluster (greenish background). These keywords are fragments of the emerging themes that have been preprocessed and tokenized. Some words integrate different categories within the theme (Fig. 5).

**Fig. 6:**
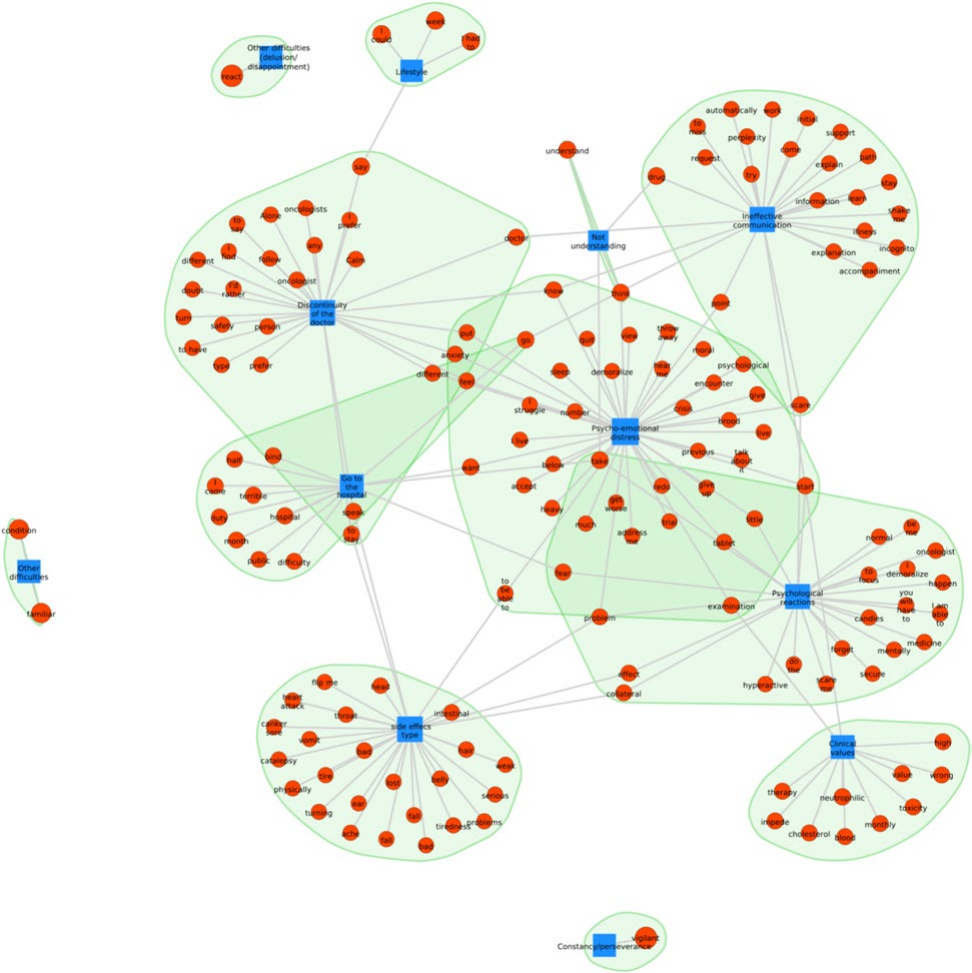
Network analysis showing the connection and recurrence between the main categories (blue) and the keywords (red) in theme 2

The central category in theme 2 related to barriers to adherence to OATs is psycho-emotional distress, which means symptoms of stress, anxiety, and depression arising from treatment, precisely elements categorized as discontinuity of doctor in charge of the patient care, types of side effects, psychological reactions, clinical results, hospital visits, lack of understanding about the treatment and poor communication with doctors (Fig. 6). All these elements were characterized as barriers to adherence to oral treatment and were directly related to patients’ psycho-emotional distress. A deeper analysis of the integration between these categories reveals the keywords that provide more detail about the emerging themes related to barriers to adherence.

**Fig. 7:**
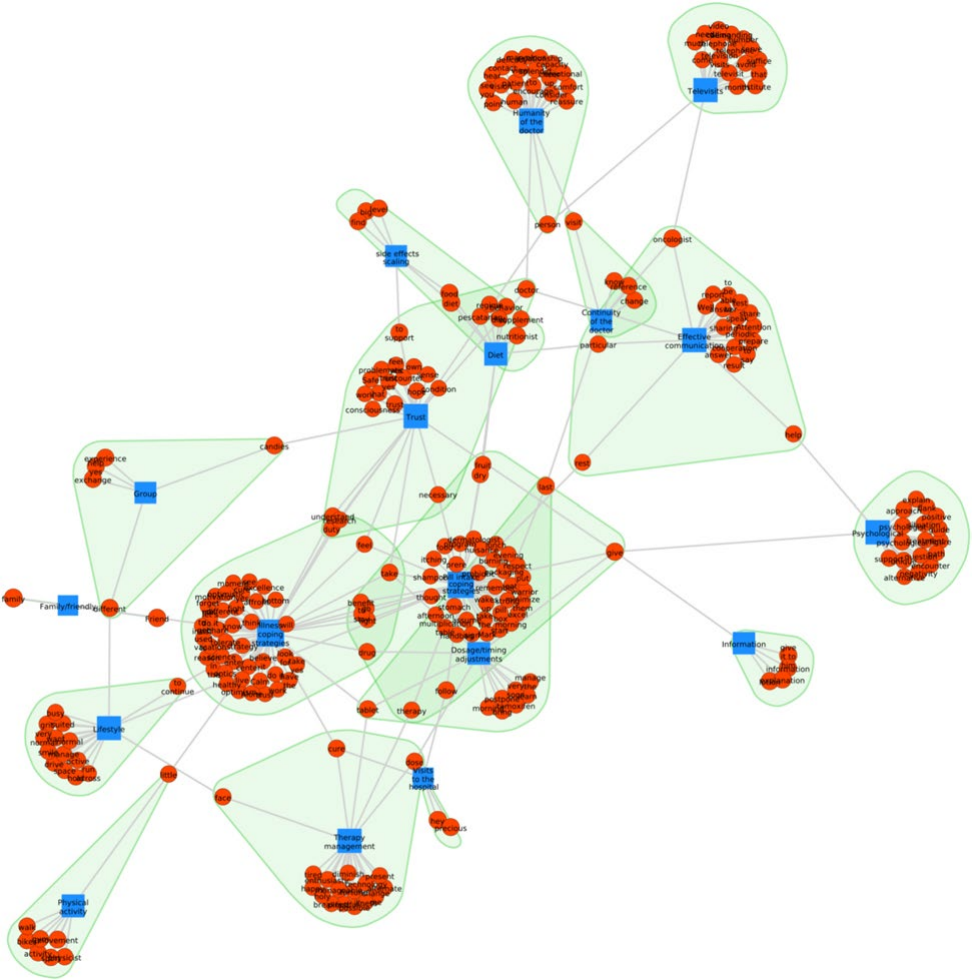
Network analysis showing the connection and recurrence between the main categories (blue) and the keywords (red) in theme 3

The central categories in the theme of facilitators of adherence to OATs (Theme 3) were strategies related to coping with the disease and the adversities arising from therapy, with the central element of adjusting the dose or time of taking the medication. Another category central to the theme was trust in the doctor, which was directly related to the continuity of the doctor, effective communication, the doctor’s humane behavior toward the patient, and reassessment of side effects. This last category had direct associations with diet advice and the possibility of accessing relevant and reliable information about the OAT and its management. Several peripheral categories were also crucial for the topic because they relate to central categories. This was the case for support groups, lifestyles, and remote consultations (telemedicine) (Fig. 7).

**Fig. 8:**
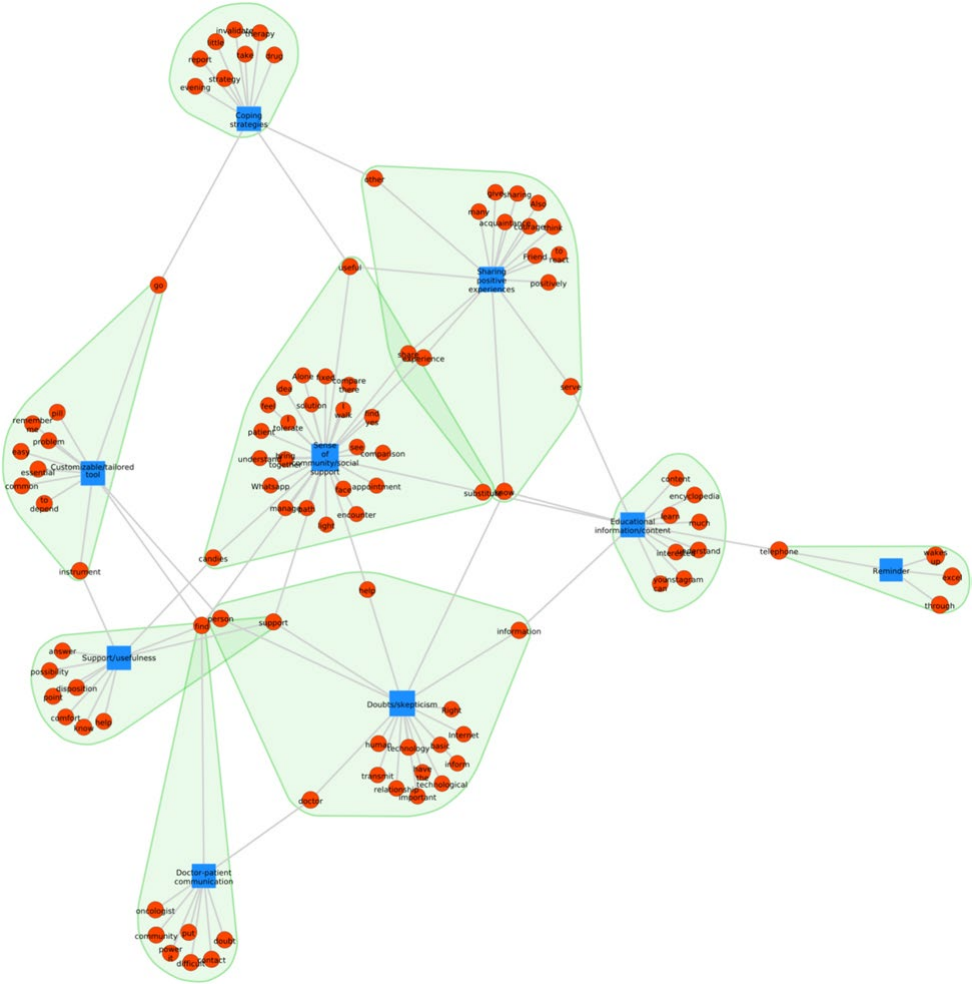
Network analysis showing the connection and recurrence between the main categories (blue) and the keywords (red) in theme 4

The network analysis in Fig. 8 illustrates patients’ perceptions of using technology as a facilitating resource for oral treatment adherence (theme 4). The central category in this theme was the sense of community and social support, which refers to the sense of belonging that patients experience when they participate in support groups with other patients having the same disease. Patients understand these virtual communities as an essential strategy for sharing positive experiences, which can be helpful as a form of support and a relevant way to access information and educational content. However, doubt and skepticism about the usefulness of technological tools for treatment adherence were issues present in this theme. The categories of coping strategies and patient-doctor communication, which were relevant in the network analysis of theme 3 (facilitators—Fig. 7), were peripheral in this theme, suggesting that patients may not see the technological tools as a facilitating resource for oral treatment to date.

### Sentimental analysis

**Fig. 9:**
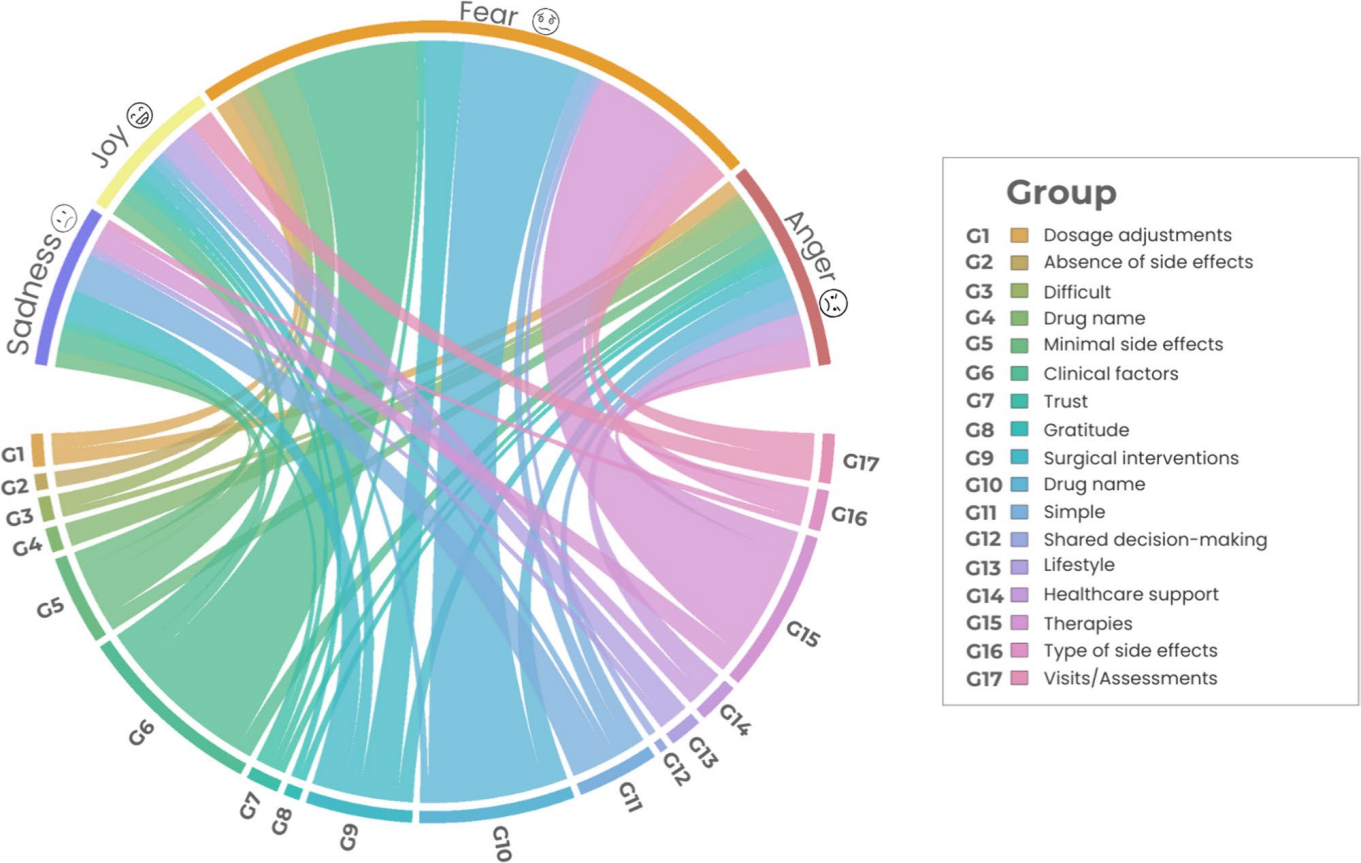
Circular relationship between emotions and category for theme 1

Fear was the most common emotion observed in responses to questions about the OAT experience. This emotion was particularly associated with the categories of “therapy,” “medication,” and “patient clinical factors” (Fig. 9). On the other hand, joy was the least expressed emotion, especially in the categories central in the network analysis for theme 1 (Fig. 5), appearing in the context of simplicity of treatment and trust in the chosen treatment.

**Fig. 10:**
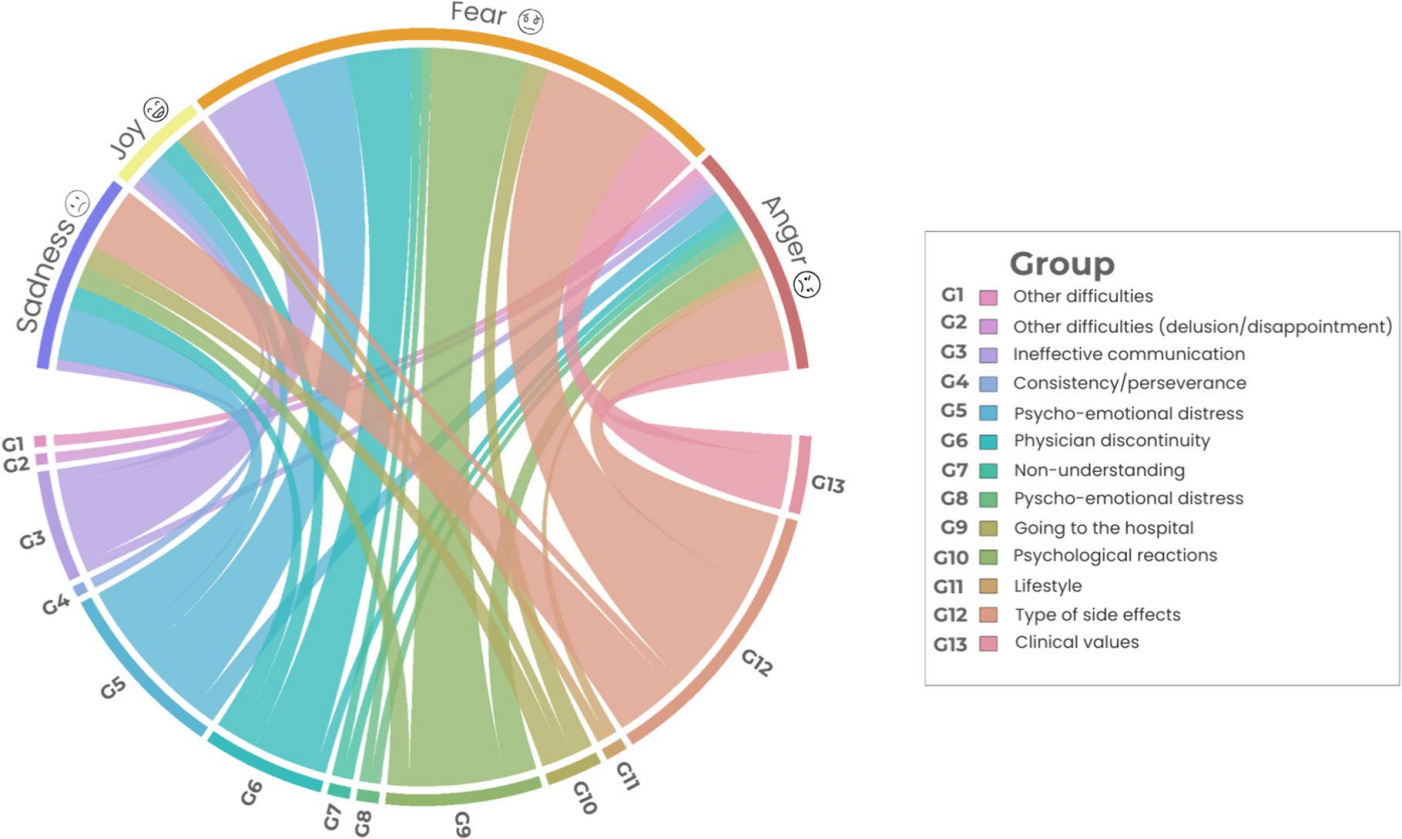
Circular relationship between emotions and category for theme 2

For theme 2, which dealt with existing barriers to adherence, the most predominant emotion was fear (Fig. 10). This emotion was particularly present for problems related to clinical values, side effects, and issues associated with the doctor-patient relationship, specifically ineffective communication, and discontinuity of the doctor in charge of the patient care. Besides, feelings of anger and sadness were frequently expressed in the category of types of side effects, highlighting the importance of this aspect as a barrier to adherence to oral treatment.

**Fig. 11:**
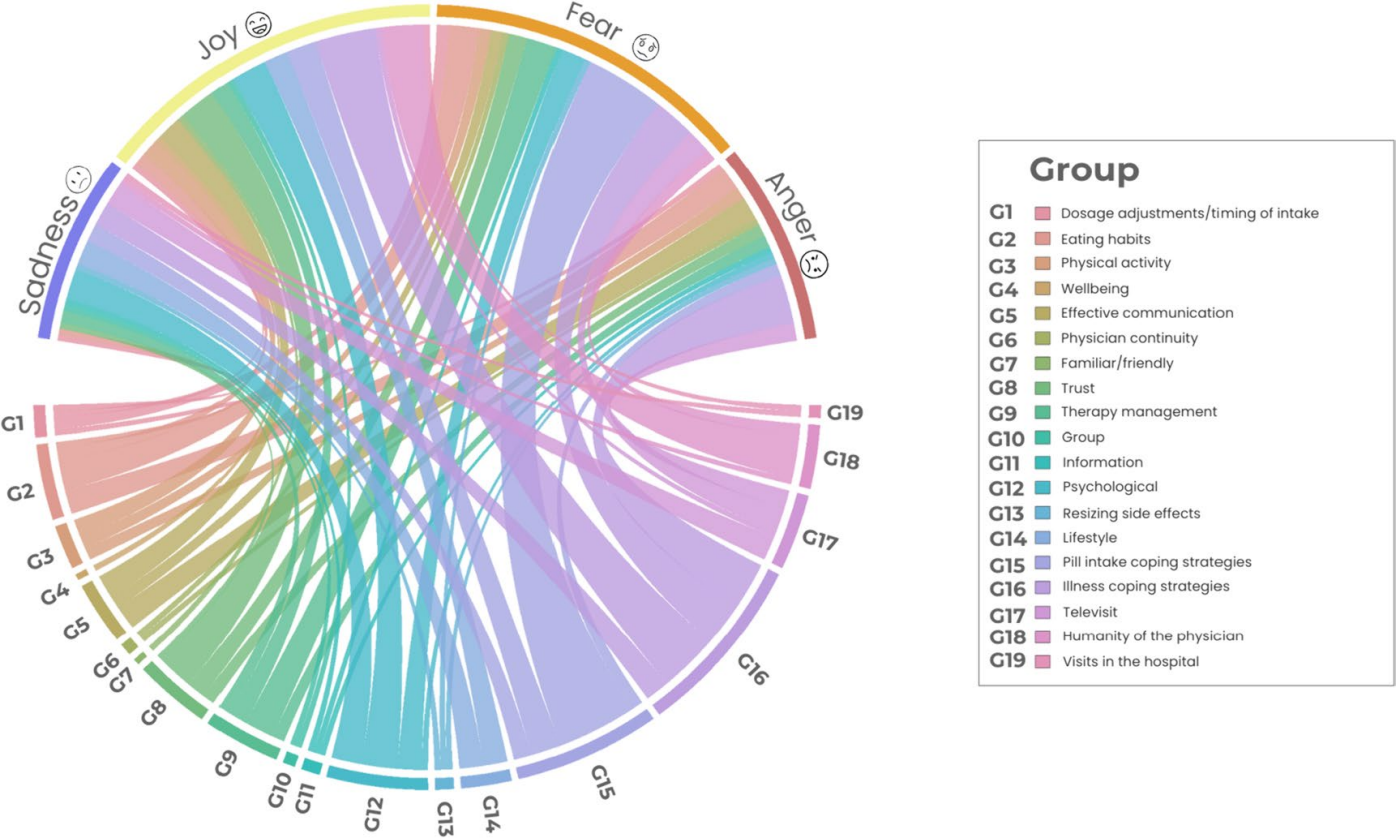
Circular relationship between emotions and category for theme 3

Compared to the other themes, theme 3, which addressed facilitators of treatment adherence, showed a decrease in feelings of fear and increased expressions of joy, which were balanced (Fig. 11). Within this theme, joy was closely related to nurturing thoughts about coping with cancer daily. In addition, joy was experienced when mentioning the physician’s humane dimension in treating patients.

**Fig. 12:**
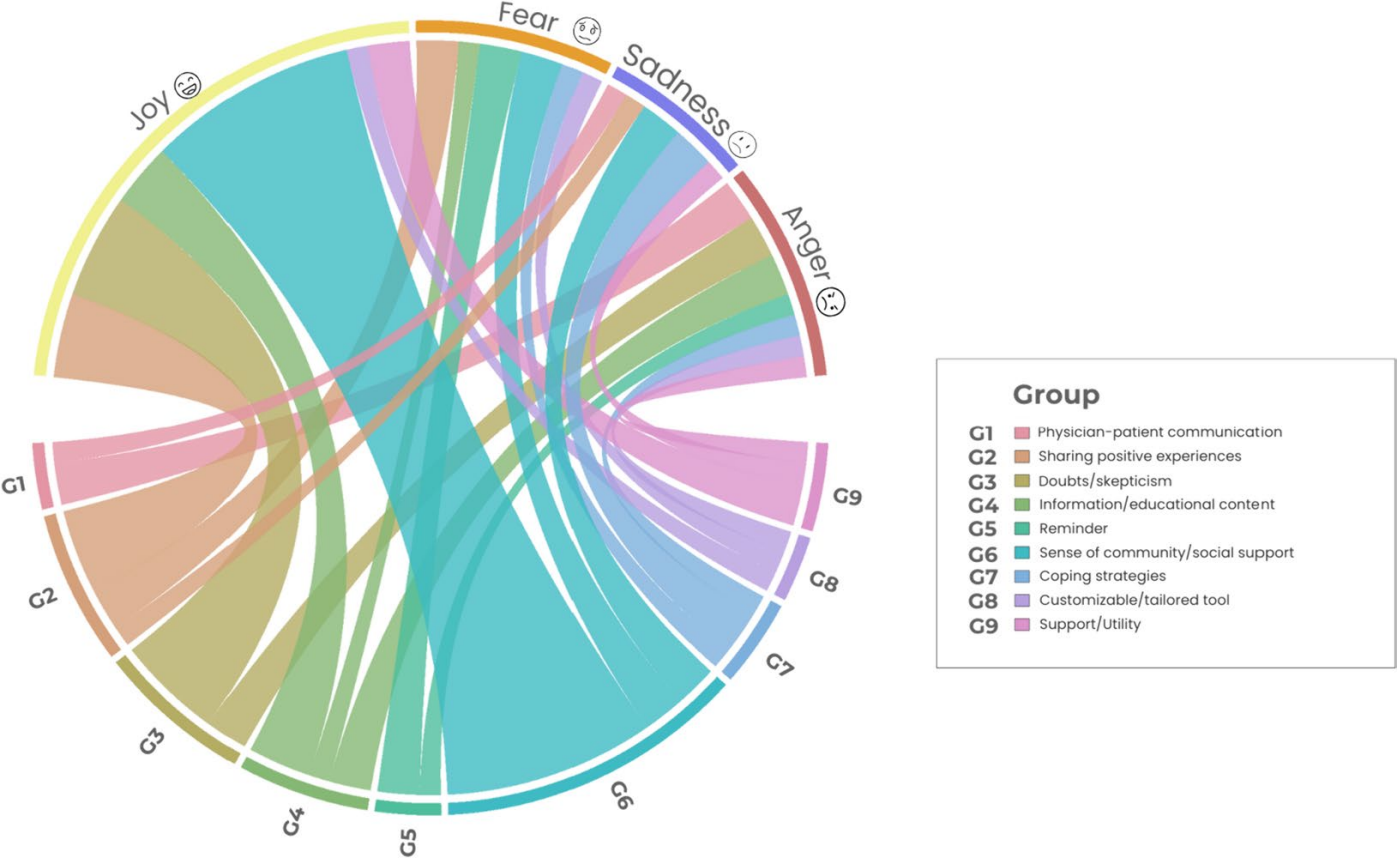
Circular relationship between emotions and category for theme 4

Regarding patients’ perceptions of the use of technology as an adherence support strategy, the most predominant emotion was joy (Fig. 12), primarily related to the central category of this theme revealed in the network analysis (Fig. 8): sense of community and social support. Despite doubts and skepticism about the theme of technological resources, they were not associated with negative emotions such as fear and sadness but rather with joy. This emotion was also widely expressed in narratives that included sharing experiences with other patients in the same condition and acquiring information and educational content (Fig. 12). Categories not associated with the emotion of joy in this theme but present in theme 3, facilitators of adherence, were coping strategies and doctor-patient communication (Figs. 7 and 11). Although these elements were considered facilitators of oral treatment adherence, they were not associated with the emotion of joy in the context of technological resources, demonstrating less impact of technological tools in the patient-doctor relationship.

## Discussion

Our results emphasized the complexity of the adherence to OATs in MBC disease, highlighting the centrality of a set of internal and external determinants related both to the specificity of the metastatic breast cancer disease and its treatments, but also psychological and relational aspects with healthcare providers. Further, they provide some input about the features that *e*Health technologies should have to foster adherence and psychological well-being.

Considering the results retrieved from the Wordcloud and network analysis, the type of side effects and relationship with the clinician affect adherence. The side effects caused a significant physical and mental load that comprised health-related QoL and daily life activities (e.g., job, family duties, and hobbies). Other studies (for example, [34, 35] have reported similar results, underlining the burden felt by patients diverging from patients with localized disease due to the complexity of the clinical protocol and its variability, as well as the important side effects experienced [35].

Otherwise, the type of relationship with the clinicians is seen by MBC patients as a resource and a roadblock. More specifically, ineffective communication and continued physician changing through clinical follow-ups hurt adherence behavior. In contrast, physicians might be a strong resource when they can be in touch with the patients and show feelings of humanity toward the patient and their condition. We argue that physicians who build a good relationship with their patients can also recognize their patients’ needs and difficulties and find a way to solve them. This late datum is coherent also with the sentiment analysis underscoring that MBC patients experience fear for issues related to clinical values (e.g., test results and breast cancer markers) and for issues related to the doctor-patient relationship, specifically ineffective communication, and discontinuity of the doctor in charge of the patient care. Coherently, joy was experienced when mentioning the physician’s humane dimension in treating patients. Besides, feelings of anger and sadness were frequently expressed in the category of types of side effects, highlighting the importance of this aspect as a barrier to adherence to oral treatment.

Additionally, internal determinants that might foster adherence were the type of coping strategies used to face cancer disease. More specifically, the analysis highlights a common cancer-relevant term within this theme, "diet," which refers to dietary adjustments that might help with treatment adherence to OATs, fostering overall physical well-being and counteracting the disease. Besides, joy was closely related to nurturing thoughts about coping with cancer daily.

An interesting result concerns the specific patients’ perceptions associated with the use of *e*Health technologies as aids to support adherence in clinical practice. MBC patients mentioned that remote clinical consultations (e.g., telemedicine) help bolster adherence, because it permits, for example, to discuss with the specialists possible problems or concerns and to find together strategies to overcome them or better manage side effects. Despite doubts and skepticism about the technological resources, patients did not feel emotions such as fear and sadness but joy. The joy was also widely expressed in patients’ narratives, including sharing experiences with other patients in the same condition and acquiring information and educational content. Our results also highlight the significance of integrating human elements, such as doctor-patient communication, and technological tools, such as support apps. The prominence of terms such as “support tool,” “phone,” “doctor,” and “person” in theme 4 may suggest that effective *e*Health technologies should synergize technological advancements with traditional healthcare approaches. One example of such an approach could be using risk-predictive models or other *e*Health technologies during shared decision-making sessions between physicians and healthcare providers. Although there is evidence that technology can improve communication between physicians and healthcare providers, few studies have explored using computer-based decision tools in oncology consultations [29].

MBC patients consistently highlighted how *e*Health technologies might increase the sense of community and social support, permitting them to be in touch with other patients who have experienced the same disease and have dealt with similar problems. According to recent studies, a vast portion of breast cancer patients accept technology (e.g., the Internet) for care assistance. As such, *e*Health technology is a promising aid in improving patient-physician communication. Such innovative systems may improve disease management and enhance treatment adherence [38, 39]. The healthcare system focuses on enhancing length and QoL; however, this requires continuous and personalized care coordination. Standard clinical methods struggle to facilitate long-term, flexible, and personalized support for MBC patients, worsening survival rates and QoL. The need for remote delivery of MBC interventions (i.e., patient-doctor communication, educational and informative content) has been demonstrated, and positive results have been assessed [40, 41].

### Study limitations

Despite the novel results highlighted by the current study in understanding adherence behavior in MBC patients, some limitations have been observed. Using a semi-structured interview guide to drive the focus group discussion may have affected the type of themes that arose. Even if we argue that the use of predetermined questions has permitted us to deepen better the specific issues related (the clinical context of patients’ characteristics and health history, barriers to adherence, resources to achieve adherence to oral treatment, and patients’ perceptions of *e*Health technologies to support adherence), probably the use of an open discussion without predetermined questions might have provided more emerging themes. This could have reduced the risk of anchoring to the predetermined questions proposed by the researcher (anchoring bias). Besides, to achieve a more comprehensive vision of the determinants of adherence, the informal caregiver perspective should have been considered to identify unconscious mechanisms in the patient that others might observe. Regarding the analyses conducted, it is worth noting that while word clouds and network analyses provide a useful overview of common themes and terms, they may lack the necessary depth to fully understand the complexities of patients’ experiences and the nuances of their emotional responses. Interpreting frequent words or phrases may be misleading and fail to capture the context in which they were used. These limitations should be addressed in future studies.

## Conclusions

Considering the critical impact of non-adherence on QoL and survival rate, attention should be paid to raising awareness to facilitate early recognition of factors predicting non-adherence to OATs. Results retrieved in the current study highlighted the importance of considering individual, relational aspects and side effects as potential predictors affecting medical adherence in MBC. These variables should be considered when risk-predictive models of non-adherence are designed and employed in clinical practice. Our results highlight the potential of new technologies to improve community and social support, as well as possible applications in treatment adherence, despite some skepticism about their effectiveness. Finally, *e*Health technology might help patients when they can connect with other patients in similar conditions and share experiences.

## Data Availability

The data are available from the authors upon reasonable request.
